# A Narrative Review of Strategies to Optimize Nutrition, Feeding, and Growth among Preterm-Born Infants: Implications for Practice

**DOI:** 10.1016/j.advnut.2024.100305

**Published:** 2024-09-21

**Authors:** Faith E Bala, Katlyn E McGrattan, Christina J Valentine, Sudarshan R Jadcherla

**Affiliations:** 1 The Innovative Infant Feeding Disorders Research Program, Nationwide Children’s Hospital, Columbus, OH, United States; 2 Center for Perinatal Research, The Research Institute at Nationwide Children’s Hospital, Columbus, OH, United States; 3 Department of Speech Language Hearing Science, University of Minnesota, Minneapolis, MN, United States; 4 Department of Pediatrics, Division of Neonatology, Banner University Medical Center, The University of Arizona, Tucson, AZ, United States; 5 Division of Neonatology, Nationwide Children’s Hospital, Columbus, OH, United States; 6 Division of Pediatric Gastroenterology, Hepatology, and Nutrition, Department of Pediatrics, The Ohio State University College of Medicine, Columbus, OH, United States

**Keywords:** neonatology, infant and young child feeding, nutritional assessment, growth assessment, nutritional management, low- and middle-income countries, preterm infant, resource-limited settings, low birth weight infant

## Abstract

Preterm birth is the leading cause of neonatal and under-5 mortality globally, and healthcare-related burden and nutrition-related morbidities are unsustainable, particularly in resource-limited regions. Additionally, preterm infants are susceptible to multiple adverse outcomes including growth faltering, suboptimal neurodevelopment, and multisystemic morbidities. Maturation, healing, repair, and restoration to normalcy in preterm-born infants require optimizing nutrition; only then, prognosis, growth, neurodevelopment, and overall quality of life can improve. In this article, we discuss the various evidence-based feeding and nutritional strategies that can be applicable even in resource-limited settings, where resources and infrastructure for advanced neonatal care are limited. This article addresses nutrition, feeding strategies, and growth monitoring in the neonatal intensive care unit and at discharge to optimize nutrition, growth, and development.

**Statement of Significance**

Nutritional and growth assessment, as well as optimizing nutrition in preterm-born infants, is a significant problem worldwide. We reviewed pertinent recent literature and provided up-to-date information for interdisciplinary teams including dietitians, nurses, feeding therapists, and physicians.

## Introduction

Globally, an estimated 13.4 million infants are born preterm (before 37 weeks of gestation) with the highest prevalence occurring in low- and middle-income countries [1], and complications from preterm birth are the leading cause of neonatal and under-5 mortality [2]. The provision of adequate nutrition is a cornerstone in the care of these infants as it directly impacts their prognosis, growth, neurodevelopment, and overall quality of life [[3], [4], [5]]. The overall goal of feeding the preterm infant, which is widely accepted by clinicians and health professionals globally, is to provide nutrients “to approximate the rate of growth and composition of weight gain akin to that of a typical fetus of the same postmenstrual age (PMA), and to maintain normal concentrations of blood and tissue nutrients while ensuring satisfactory functional development” [6,7]. To help clinicians and health professionals meet this goal, several nutrient recommendation guidelines are available for preterm infants receiving parenteral or enteral nutrition during their neonatal intensive care unit (NICU) stay [8]. However, the nutritional requirements are unique to the individual fetus ex utero owing to diverse pathophysiology, metabolic needs, and neuroendocrine regulation [9]. Thus, clinicians and health professionals caring for these infants must recognize the need for individualization and changing pathophysiology.

Physiological immaturity, feeding intolerance, and prematurity-related complications, such as necrotizing enterocolitis (NEC), sepsis, patent ductus arteriosus (PDA), intraventricular hemorrhage (IVH), and bronchopulmonary dysplasia (BPD), create significant challenges in ensuring the adequate delivery of nutrients to meet the needs of preterm infants [[10], [11], [12]]. As such, preterm infants experience significant nutritional and growth deficits that can be isolated at a given time or continuous [[13], [14], [15]]. Nutritional and growth deficits during NICU stay and beyond in preterm infants are associated with adverse neurodevelopmental outcomes [3,16]. Furthermore, nutritional and growth deficits during early infancy and rapid catch-up growth periods may predispose preterm infants to long-term adverse metabolic and cardiovascular outcomes [17,18]. Childhood and adult obesity and associated risks among preterm-born infants are also long-term concerns [19].

Optimizing the nutritional support of preterm infants during their NICU stay has been shown to reduce accumulated nutrient and growth deficits [4,5]. Continuous provision of adequate nutrition postdischarge will also prevent growth faltering and excessive catch-up growth [20]. Numerous strategies have been utilized to optimize the nutritional support of preterm infants [8]. However, some strategies may not be feasible in resource-limited settings where access to advanced neonatal care and specialized feeding may be limited or unavailable. For example, prompt commencement of parenteral nutrition when estimated energy and nutrient requirements cannot be safely and adequately provided enterally is an important strategy for optimizing the nutritional support of preterm infants and is recommended by numerous expert groups including the American Society of Parenteral and Enteral Nutrition and the European Society for Pediatric Gastroenterology, Hepatology, and Nutrition (ESPGHAN) [8,21]. However, the infrastructure and resources needed for the safe preparation, administration, and monitoring of parenteral nutrition may be lacking in numerous resource-limited settings [22]. Nonetheless, several evidence-based strategies for optimizing nutrition support in such settings are available.

## Status of Knowledge

### Literature search

A literature search was conducted on PubMed, Scopus, and Cochrane Library to identify relevant articles on the nutritional management of preterm infants. Literature searches were conducted up to April 2024, with no time restriction for the publication date. We considered guidelines, systematic reviews, nonsystematic reviews, randomized controlled trials (RCTs), and observational studies. Reference lists of articles were also manually searched for additional relevant articles. Articles were critically evaluated for relevance to each area of interest for this review. Studies that focused on full-term infants and other pediatric patients, studies that were not available in English, and studies for which full text was unavailable were excluded.

### Nutrition in the NICU

#### Nutrient recommendations for preterm infants

The book “Nutritional Care of Preterm Infants: Scientific Basis and Practical Guidelines” [23] provides nutrient intake recommendations for preterm infants with a birth weight ≤1500 g (Table 1). Similar nutrient recommendations are also available from ESPGHAN for infants with a birth weight ≤1800 g (Table 1) [24]. The recommended intake values aim to fulfill the nutrient requirements of all growing and stable very low birth weight (LBW) preterm infants who are receiving nutrition enterally. Nonetheless, individual nutrient requirements are variable and influenced by factors including “gestational age, PMA, birth weight, current weight, weight gain rates, comorbidities, and other contributing factors” [23,24]. Table 2 provides a list of critical nutrients and their functions in preterm infants [[24], [25], [26], [27], [28], [29], [30], [31], [32], [33], [34], [35], [36], [37], [38], [39], [40], [41], [42], [43], [44]].

**TABLE 1:** Enteral nutrient recommendations for preterm infants

Nutrient per kg/d	Koletzko et al., 2021 [23]	ESPGHAN, 2022 [24]
Fluid, mL	135–200	150–180 (135–200)^1^
Energy, kcal	110–130	115–140 (−160)^1^
Protein, g	3.5–4.5	3.5–4 (−4.5)^1^
Lipids, g	4.55–8.1	4.8–8.1
Linoleic acid, mg	385–1540	385–1540
α-Linolenic acid, mg	>55	≥55
DHA, mg	ns	30–65
EPA, mg	ns	<20
ARA, mg	ns	30–100
DHA/ARA ratio	0.5–1	ns
Carbohydrate, g	11–13	11–15 (−17)^1^
Sodium, mg [23], mmol [24]	69–115 (–184)^1^	3–5 (−8)
Potassium, mg [23], mmol [24]	78–195	2.3–4.6
Chloride, mg [23], mmol [24]	105–177 (–284)^1^	3–5 (−8)^1^
Calcium, mg [23], mmol [24]	120–220	3–5
Phosphorus, mg [23], mmol [24]	70–120	2.2–3.7
Magnesium, mg [23], mmol [24]	8–15	0.4–0.5
Iron, mg	1–3	2–3 (−6)^1^
Zinc, mg	2–3	2–3
Copper, μg	120–230	120–230
Selenium, μg	7–10	7–10
Manganese, μg	1–15	1–15
Fluoride, μg	ns	ns
Iodine, μg	10–55	11–55
Chromium, μg	0.03–2.25	0.03–2.25
Molybdenum, μg	0.3–5	0.3–5
Thiamine, μg	132–275	140–290
Riboflavin, μg	200–430	200–430
Niacin, mg	1.1–5.5	1100–5700
Pantothenic acid, mg	0.6–2.1	0.6–2.2
Pyridoxine, μg	66–275	70–290
Cobalamin, μg	0.12–0.6	0.1–0.6
Folic acid, μg	22–100	23–100
L-Ascorbic acid, mg	16.5–41	17–43
Biotin, μg	3.3–15	3.5–15
Vitamin A, IU	1332–3330	1333–3300 (400–1000)^1^
Vitamin D, IU	400–1000	400–700 (<1000)^1^
Vitamin E, mg α-TE	2.2–11	2.2–11
Vitamin K1, μg	4.4–28	4.4–28
Choline (total free and bound), mg	≥33	ns

Abbreviations: α-TE, α-tocopherol; ARA, arachidonic acid; DHA, docosahexaenoic acid; EPA, eicosapentaenoic acid; IU, international units; ns, not stated.

1 Values within parentheses denote ranges or upper intake levels that may occasionally be required in standard clinical practice under specific conditions.

**TABLE 2:** Select nutrients and their functions in preterm infants

Nutrient	Function
Protein [24,[25], [26], [27]]	Weight gain; Lean body mass accretion; Linear growth; Repair and regeneration; Neurodevelopment; Immune competence
Vitamin A [[28], [29], [30]]	Anti-inflammation; Immune competence; Growth; Vision; Lung growth; Integrity of the respiratory tract epithelial cells; Neurodevelopment
Vitamin D [28,31,32]	Immune competence; Respiratory function; Bone health; Neuromuscular function
Iron [28,31,33,34]	Neurodevelopment; Iron status
Zinc [28,[35], [36], [37]]	Energy, protein, carbohydrate, lipid, and nucleic acid metabolism; Neurodevelopment; Growth; Wound healing and tissue maintenance
Selenium [[38], [39], [40]]	Protection against free radical damage; Thyroid function; Immune function; Metabolic functions; Cardiovascular health
Copper [41]	Neurodevelopment; Essential functioning of organs; Protection against oxidative damage
Chromium [28,42]	Glucose metabolism/tolerance
Molybdenum [43]	Cofactor for multiple enzymes essential for oxidation and reduction
Chromium [28,42]	Glucose metabolism/tolerance
DHA [43,44]	Neurodevelopment; Retinal/visual development; Growth and modulation of immune functions

#### Fluid and electrolyte considerations

Preterm infants vary greatly in their needs for fluids owing to differences in their body sizes, proportions, surfaces, and skin characteristics [24]. Preterm infants with lower gestational age and birthweight exhibit considerably higher extracellular fluid loss in the initial days after birth. Insensible water loss is also higher because of their high surface area to body mass ratio and immature, water-permeable skin. Furthermore, factors such as birth asphyxia, respiratory distress syndrome, BPD, PDA, NEC, and treatment conditions (phototherapy, radiant warmers) affect an infant’s need for fluids. The optimal fluid intake may also vary based on macronutrient intakes, with higher protein intakes probably necessitating higher fluid intakes [24]. Preterm infants also have immature renal function with limited renal concentration ability and excretory capacity [45]. Appropriate and safe administration of fluids and electrolytes must consider all these variables.

On the first day of life, fluid volumes are normally between 70 and 100 mL/kg/d with more immature infants requiring even more to account for fluid losses depending on the degree of immaturity [46]. Initial fluids are provided as parenteral fluids, preferably as parenteral nutrition, in newborns who are not yet ready for enteral feeding. However, parenteral nutrition may not always be possible in resource-limited settings, and infants in such settings are given intravenous fluids containing glucose and electrolytes [47]. Daily fluid increases usually range from 20 to 40 mL/kg/d in more mature infants and up to 30 to 60 mL/kg/day or higher in more immature patients and depend on input and output considerations [48]. As infants that were started on parenteral fluids become capable of enteral feedings, the parenteral fluid volumes should be tapered down accordingly so that total fluid intake does not exceed prescribed requirements. Full fluid intake is reached when intake ranges from 135 to 200 mL/kg/d (Table 1) [24,48]. To prevent renal impairment in some infants, fluid intake as low as 135 mL/kg/d may be deemed safe and adequate to maintain body homeostasis provided nutritional needs can be adequately satisfied [24]. Extra caution is necessary particularly in infants with BPD or PDA if volumes of up to 200 mL/kg/d are administered [24]. Regarding electrolytes, sodium in particular, intake should be low in the initial phase when infants lose extracellular fluids and should range from 1 to 3 mmol/kg/d. Giving such small amounts rather than being sodium-free reportedly reduces the risk of hyponatremia in the first few days of life [48]. Once extracellular fluid contraction is complete and the infant is in the stable phase of growth (when full enteral feeding is attained), intakes are from 3 to 5 mmol/kg/d of sodium [48].

### Feeding strategies in the NICU

#### Human milk – mother’s own milk or donor human milk

The WHO together with other expert groups recommends mother’s own milk (MOM) as the first feed of choice for all infants including preterm infants [49,50]. There is strong and consistent evidence from observational studies that feeding MOM to preterm infants, either exclusively or in combination with other milk, as opposed to only infant formula feeding, is associated with reduced incidence of NEC [51]. When MOM is unavailable, donor human milk (DHM) is recommended as the next option during acute care [49,50]. Evidence from a Cochrane review [52] including studies from high-income settings indicates that feeding preterm infants or LBW infants with DHM solely or in combination with MOM rather than infant formula reduces the incidence of NEC and feeding intolerance. DHM, however, resulted in a slower growth rate in terms of weight, length, and head circumference during the in-hospital period, although evidence on long-term growth outcomes is limited [52]. Inequalities exist in access to DHM because safe and affordable milk-banking facilities are not evenly distributed globally and are unavailable in most resource-limited settings [53,54]. Thus, the potential for DHM in the absence of MOM to reduce the incidence of prematurity-related morbidities remains unexplored in such settings. Although the practice of wet nursing and informal milk sharing is common in many resource-limited settings [55], there is currently no data on its safety, and a recommendation for this practice is not available.

#### Human milk fortification

The levels of some key nutrients, most notably protein, calcium, and phosphorus, in both MOM or DHM do not adequately meet the nutritional needs of preterm infants, in particular very preterm (<32 weeks of gestation) and extremely (<28 weeks of gestation) infants [56]. Nutrients are also limiting due to the low feeding volumes of preterm infants compared to the term infant. To augment the nutritional content of MOM or DHM and to better support the nutrient needs of this subset of preterm infants, multicomponent fortifiers may be considered [49]. They provide additional protein, energy, vitamins, and minerals, and their addition to MOM or DHM is crucial for preventing postnatal growth faltering and micronutrient deficiency, promoting bone mineralization, and optimizing neurodevelopment [57]. Available evidence from a Cochrane review [58] indicates that fortification of MOM or DHM with multicomponent fortifiers resulted in a modest increase in in-hospital weight, length, and head circumference with no evidence of harm in very preterm and very LBW infants. There is no consensus on the best time to start MOM or DHM fortification. Typically, multicomponent fortifiers are added to MOM or DHM when ≥100 mL/kg/d of enteral feeding or when full enteral feeding, typically 150 mL/kg/d is achieved [59].

Multicomponent fortifiers are derived from human or animal milk, and they are commercially available as liquids or powders. Recent interest has been the use of a human milk-derived fortifier, as it promotes an exclusive human milk diet; however, the cost of using human milk-derived multicomponent fortifiers is considerably high even in high-income settings, and the currently available evidence does not support its recommendation over animal-based fortifiers. A Cochrane review [60] identified only 1 trial, and there was no difference in NEC, feeding intolerance, late-onset sepsis, or morbidity between the use of human milk-derived fortifier or bovine-derived fortifier. Liquid fortifiers are sterile, as opposed to powdered fortifiers, which are susceptible to contamination by microbes such as *Cronobacter sakazakii*. However, liquid fortifiers displace significant amounts of human milk, potentially reducing the dose-dependent protective benefits of human milk. Although liquid fortifiers are recommended and usually utilized in high-income settings, their use in resource-limited settings is limited [61]. In resource-limited settings, fortifiers may be unavailable or too expensive, and nutrient-enriched preterm formulas may be used to fortify human milk instead [62,63]. Irrespective of the type of fortifier used, institutions should strive for best practices using human milk rooms for mixing [64] and trained technicians to reduce errors and enhance safety [65].

Although improvements in the nutrient profile of multicomponent fortifiers have been made over the past few decades, they are still not adequately suited to meet the recommended intake for all macronutrients and micronutrients. One report stated that after standard fortification of human milk assuming a feed intake of 180 mL/kg/d, the recommended intakes for protein, vitamin A, vitamin D, and iron were still not met [66]. The standardized approach to fortification also assumes that MOM or DHM have a consistent composition, which is not the case as there is large inter- and intra-individual variability [67]. Single-nutrient fortifiers have been used in conjunction with multicomponent fortifiers to individualize MOM or DHM fortification so that actual intake closely matches recommended intakes [[68], [69], [70]].

There are 2 methods for individualizing the fortification of MOM or DHM. Targeted fortification adds macronutrients to human milk to help reach a target composition after a baseline analysis of its composition using a human milk analyzer [71]. Adjustable fortification adds nutrients based on the preterm infant protein status determined by blood urea nitrogen (BUN) [69]. Although BUN is influenced by renal function and hydration status, its value is proportional to protein intake and responds rapidly to changes in protein intake [72]. A target BUN range of 10 to 16 mg/dL has been proposed with BUN <10 indicating protein insufficiency [69]. According to a Cochrane review [73], standard fortification in comparison to individualized (either targeted or adjustable) resulted in increases in weight, length, and head circumference velocities during the intervention period in preterm infants of <32 weeks of gestation. Currently, there is no consensus about the best practice: adjustable or targeted. Both approaches are expensive and may not be feasible in resource-limited settings.

The concentration of some nutrients in MOM such as vitamins A, B1, B2, B6, B12, C, and D, essential fatty acids, choline, selenium, and iodine can be dependent on the maternal diet [74,75]. Hence, dietary intake of these nutrients should be reviewed with the mother, and maternal dietary education and counseling to improve the delivery of these nutrients to the infant should be provided by the nutrition support team [76]. Unfortunately, donating mothers often do not receive targeted nutritional counseling, and compounding the problem is that the milk is collected at a late lactational stage ranging from 4 to 12 mo compared to fresh preterm milk [77]. This results in even more significant reductions in protein, fats, sodium, vitamins, iron, and zinc [78]. It may also be advantageous for mothers to continue taking their prenatal vitamins [76]. This is especially important in resource-limited settings where food and nutrition insecurity are widespread. Mothers bringing randomly pumped milk to the NICU could inadvertently have little fat concentration in the milk; thus, counseling her on pumping hind milk can significantly improve fat intake and growth velocity of preterm infants [79]. Hindmilk is the milk expressed after ∼2 min of pumping, and it is richer in fat and calories than foremilk [80]. The mother can collect it in a separate bottle and bring it to the unit for feeding the infant. Infant fat intake and growth are significantly improved in this practice [79,81]. Commercially available human milk cream derived from DHM is also available and can accelerate weight gain [82]. It must be acknowledged that increasing the energy content alone without consideration for protein and other nutrients can disturb the protein-to-energy ratio and inadvertently result in a less-than-optimal body composition [83]. The administration of protein at a rate of 2.8 to 3.6 g/100 kcal when energy and protein intakes are within the recommended ranges permits the accretion of fat-free mass and fat mass in optimal proportions and may have ramifications for long-term health [83,84].

#### Infant formulas

When MOM and DHM are unavailable, nutrient-enriched formulas specifically intended for preterm infants may be considered for very preterm infants [49]. These formulas are energy-enriched usually up to about 80 kcal/100 mL with variable enrichment of other nutrients, and they aim to provide nutrients that allow accretion at intra-uterine accretion rates. In contrast, standard formulas are designed for term infants and are formulated to mimic the composition of mature human milk. Available evidence in very preterm infants indicates that compared to standard formulas, nutrient-enriched formulas are associated with in-hospital increases in weight and head circumference rates and psychomotor developmental scores at 18 mo with no effect on bone mineralization or long-term growth and development [85]. In most resource-limited settings, standard powdered infant formulas designed for term infants may be the only option for very preterm infants when MOM or DHM is not available, putting the infants at a nutritional disadvantage [61]. Nutrient-enriched formulas are available as either liquids or powders. Because liquid formulas are sterile, they should be used whenever it is possible and feasible to do so. When powdered formula is used, it must be prepared hygienically by ensuring proper handwashing, clean utensils, clean and boiled water to reconstitute the formula, and heating of reconstituted formula at the point of use [61].

#### Nutritional supplements

Nutritional supplements, available as single- or multiple-micronutrient preparations can be given separately from MOM or DHM to preterm infants. If the infant is ingesting fortified human milk (MOM or DHM) or nutrient-enriched preterm formula, most micronutrients are adequately provided at volumes ≥150 mL/kg and additional supplementation may not be needed. Thus, the WHO has only made recommendations for supplementation with iron, zinc, vitamin A, and vitamin D in human milk-fed preterm infants who are not receiving these nutrients from other sources (Table 3) [49]. With regard to all these nutrients, data on the timing of initiation and duration of supplementation is limited; hence, the WHO recommends that supplementation may begin when enteral feeds are well established and may continue until the infant adequately receives these nutrients from another source [49]. It must be acknowledged that adequate intakes may not be guaranteed even in infants receiving fortified feeds, and additional supplementation may be necessary.

**TABLE 3:** Evidence-based practical nutritional and feeding strategies for all preterm infants

**Human milk** • Mother’s own milk (MOM) is the preferred first choice. • When MOM is not available, DHM may be considered in acute care settings.
**Multicomponent fortification of human milk** • Not routinely recommended for all preterm infants. • Consider using it for very preterm infants who are fed MOM or DHM.
**Nutritional supplements for human milk-fed infants.** • Enteral iron at a dose of 2–4 mg/kg/d is required. • Enteral zinc at a dose of 1–3 mg/kg/d may be considered. • Enteral vitamin D at a dose of 400–800 IU may be considered. • Enteral vitamin A supplementation at a dose of 1000–5000 IU may be considered for very preterm infants.
**Infant formula** • Consider nutrient-enriched preterm formula for very preterm infants when MOM and DHM are not available.
**Feeding schedule** • Until discharge, hospitalized preterm infants born before 34 weeks of gestation may be fed on schedule rather than responsively.
**Feeding initiation** • Initiate feeding as early as possible, ideally starting from the first day after birth. • Infants who are stable to breastfeed should be put to the breast as soon as possible after birth.
**Feeding progression** • Feed volumes can be accelerated by daily increments of up to 30 mL/kg in preterm infants who require an alternative feeding method to breastfeeding.
**Duration of exclusive breastfeeding and postdischarge nutrition** • Preterm infants should be exclusively breastfed until 6 mo of age.

Abbreviations: DHM, donor human milk; MOM, mother’s own milk. Source: [49]

The demand for iron is high in preterm infants because of “low stores at birth, early onset erythropoiesis, frequent blood draws, and rapid catch-up group” [28]. A systemic review [86] found that enteral iron supplementation at a dose of 2 to 4 mg/kg/d in human-fed preterm and LBW infants led to increases in length and hemoglobin levels and reduced the prevalence of anemia. Supplementing iron did not increase the risk of severe morbidities such as late-onset sepsis, BPD, and retinopathy of prematurity (ROP) [86]. The cost involved with iron supplementation is marginal, and it can be readily administered in resource-limited settings [61]. Iron supplements are commonly provided to LBW and preterm infants as oral liquid solutions and can be administered to the infant using droppers or syringes [49].

The levels of vitamin D in human milk are low, and many preterm infants are not born with sufficient vitamin D stores [87]. A systematic review [88] found that supplementing vitamin D at a dose of 200 to 800 IU/day in human milk-fed preterm or LBW infants during the first 6 mo of life increased weight, length, and head circumference and decreased the incidence of vitamin D deficiency. Vitamin D is commonly available as a component in multivitamin formulations and can be administered to the infant using droppers or syringes [49].

Preterm infants have low zinc stores and excessive endogenous losses, and human milk may not be able to meet their nutrient requirements [35]. In a recent Cochrane review [89] evaluating enteral zinc supplementation in hospitalized preterm infants receiving any type of milk (infant formula or human milk), the findings indicated that zinc supplementation reduced overall mortality and was likely linked to an improvement in short-term weight gain and linear growth. However, its impact on common morbidities of prematurity was minimal or negligible. Similarly, a recent systematic review [90] reported increased weight, length, and head circumference and decreased diarrhea with enteral zinc supplementation in human milk-fed preterm infants. Zinc supplements are often available as capsules that can be dissolved in water to yield 1 mg elemental Zn/mL [61].

Preterm infants are born with limited vitamin A stores at birth, and supplementation is thought to improve lung function [28]. A recent systemic review [91] of 4 RCTs that enrolled very preterm or very LBW infants reported that a daily dose (<10,000 IU/d) of enteral vitamin A supplementation increased the concentration of serum retinol concentration in very preterm infants but had little or no effect on mortality and various morbidities of prematurity, including BPD, pneumothorax, pulmonary hemorrhage, ROP, PDA, and periventricular leukomalacia. There were no trials in infants born ≥32 weeks of gestation or ≥1.5 kg birth weight, so no recommendations were made for those infants. “Enteral vitamin A supplementation may be considered at a dose of 1000 to 5000 IU for human milk-fed very preterm infants who are not receiving vitamin A from another source” [49]. The evidence is currently inadequate to make recommendations for other groups of preterm infants.

Supplementation with long-chain polyunsaturated fats such as arachidonic acid (ARA) and DHA has been suggested as preterm infants have enzyme immaturities that necessitate their addition [92]. These supplements are provided via preterm formula, supplements, or human milk fortifiers. Maternal supplementation of DHA should also be considered to improve concentrations in her milk [93,94]. Studies have shown that preterm infants on traditional protocols that are not supplemented with adequate DHA and ARA have measured levels significantly lower than had they stayed in utero [95], and these drops in blood levels have been associated with morbidities such as BPD [96] and ROP [97]. In addition, in a recent study of 120 preterm infants <29 weeks of gestation, a daily dose of 100 mg ARA and 50 mg DHA enhanced white matter maturation [98].

#### Feeding initiation and advancement

Early initiation of enteral feeding is recommended from the first day of life for most preterm infants including those <32 weeks of gestation [49]. Although there are concerns about potential health complications with early feeding initiation, particularly NEC, current evidence does not support these concerns. According to a systematic review [99], early initiation of enteral feeding within the first 72 h of life rather than delayed feeding initiation after 72 h significantly reduces the risk of neonatal mortality, may decrease the likelihood of sepsis, and decreases the length of hospitalization. Early initiation of enteral feeding had minimal impact on NEC, IVH, feeding intolerance, time to regain birth weight, head circumference, and length at discharge. In the included trials, initial enteral feeding volumes were minimal and ranged from 5 to 25 mL/kg/d, with only 2 studies providing infants with feed volumes >15 mL/kg/d [99]. However, a Cochrane review [100] of 6 trials of 526 preterm or LBW infants weighing between 1000 g and 1500 g at birth compared early full enteral feeding (60–80 mL/kg feeds on the first day after birth) with minimal enteral feeding (typically 20 mL/kg on the first day in addition to intravenous fluids) and found no evidence of an effect on NEC or other clinically important outcomes.

Once enteral feeding has been initiated, feed volumes may be rapidly progressed beyond minimal volumes. According to a Cochrane review [101], delaying the initiation of progressive enteral feeds after 4 d did not provide any benefits in terms of prevention of NEC, growth, infection, or mortality. A progressive volume increase of up to 30 mL/kg/d is recommended for preterm infants including those <32 weeks of gestation [49]. According to a systematic review [102], the rapid advancement of feeds by >30 mL/kg/d compared to slow advancement rates led to an almost 4-d decrease in the time taken to regain birth weight and a 3-d reduction in the length of hospitalization. Fast-feed advancement also led to a moderate decrease in the risk of mortality, NEC, sepsis, and feed intolerance. Weight or head circumference outcomes at discharge were largely unaffected by fast-feed advancement. A 2021 Cochrane review [103] compared slow advancement rates (15–20 mL/kg) and fast rates (30–40 mL/kg) in very preterm and very LBW infants and found no difference in mortality, NEC, or length of hospital stay. However, slower feed advancement moderately increased feeding intolerance and invasive infection risks. Infants with slow advancement rates took several days longer to establish full enteral feeding and regain birth weight than those with faster advancement rates.

Despite the evidence indicating that early initiation and fast progression of enteral feeding do not lead to adverse outcomes, there is significant variability across NICUs in their feeding practices. Enteral feeding practices are often influenced by local customs and the experiences of clinicians and health professionals [104]. In high-income settings, the approach to enteral feeding initiation and progression is quite conservative due to concerns for feeding intolerance, gastro-esophageal reflux, aspiration, and NEC [105]. A common strategy in such settings is to start with minimal enteral feeding within the first few days after birth and delay progressive enteral feedings until minimal volumes are well tolerated and absorbed [104]. Table 4 [[106], [107], [108]] provides a feeding protocol intended for a high-income setting. Such a strategy in resource-limited settings can result in significant growth and nutrient deficits because parenteral nutrition may be unavailable [55]. Rather, the strategies employed in resource-limited settings tend to favor early initiation and advancement of enteral nutrition in stable preterm infants [109]. It must be acknowledged that care must be taken in applying these pragmatic strategies in extremely preterm, extremely LBW (<1000 g), and unstable preterm infants including those with congenital anomalies and birth asphyxia because most trials excluded or had few infants with these conditions. Such infants may benefit from conservative approaches and feeding initiation and advancements should be based on clinical judgment.

**TABLE 4:** Feeding protocol from a high-income setting

Maturational age	Feeding milestone
Within 3 d of life	- Start with trophic feeding of 10–20 mL/kg/d and continue for 3–5 d.
By day of life 14–28 d	- Progress to full enteral feeds of ≥120 mL/kg/d. - Begin recognizing cues for hunger.
28–32 wk PMA	- Initiate nonnutritive stimulation via pacifier, pacifier dipped in milk, nonnutritive breastfeeding, and kangaroo care.
33–34 wk PMA	- First oral feeding for clinically stable infants.
36–38 wk PMA	- Full oral feeding of ≥120 mL/kg/d owing to ductal issues and lung disease.
Term equivalent age/discharge	- Ad lib oral feeding.

Abbreviation: PMA, postmenstrual age. Sources: [[106], [107], [108]]

Standardization of feeding protocol has been identified as an important approach for improving the quality of care and reducing unnecessary variations in nutritional practices within the NICU [110]. Elements that may be standardized in a feeding protocol in addition to feeding initiation and progression include what to feed, how to feed, when to fortify human milk, how to evaluate for various signs of feeding intolerance, and when to withhold enteral feeding. Implementing some or all of these elements as part of a standardized feeding protocol has been shown to improve clinical outcomes. Such outcomes include a shorter time on parenteral nutrition, a shorter time to transition to full oral feeding, a shorter length of hospital stay, improved postnatal growth, and a reduction in the incidence of NEC [106,111,112].

#### Feeding method – intragastric route

The establishment of safe oral feeding in preterm infants may be delayed because of poor coordination of sucking, swallowing, and breathing, particularly in those <32 weeks of gestation [11]. Although stimulation of suck-swallow-breathe-oral-pharyngeal-esophageal peristalsis sequences is possible with oral offerings, existing comorbidities and the current level of immaturity preclude attaining oral feeding efficiency [11]. Oral feeding also depends on the development of appropriate airway and digestive reflexes that are adaptive and protective, to ensure airway safety and appropriate oral-pharyngeal-esophageal transit. Hence, preterm infants can receive nutrition through orogastric or nasogastric tubes, utilizing either continuous or intermittent bolus delivery of milk until they are capable of fully feeding orally [113].

Nasogastric tubes are commonly preferred due to their ease of fixation, but they can partially obstruct nasal passages, potentially affecting respiratory function. This obstruction increases the work of breathing, resulting in higher energy expenditure. Consequently, this may lead to poor growth and inadequate nutrient intake. In contrast, orogastric tubes are prone to displacement, may cause local irritation, and can even stimulate the vagus nerve [113]. In a 2013 Cochrane review [113] comparing nasogastric and orogastric tube feeding, no significant difference in “the time taken to establish enteral feeding, the time taken to establish full oral feeds, the time taken to regain birth weight, weight velocity, incidence of apnea, desaturation, and bradycardia” was found.

A recent RCT [114] reported “a shorter time to regain birthweight, lower incidence of aspiration and tube displacement, a shorter time to achieve full enteral feeding” among preterm infants who were fed using a bolus nasogastric tube as opposed to a bolus orogastric tube. The study did not observe any significant differences in the incidence rates of apnea, NEC, bradycardia, oxygen desaturation, or gastric residuals between both groups. Given the currently available evidence, no method is recommended for preterm infants in both high-income and resource-limited settings, and healthcare providers may use whatever methods they prefer.

Intragastric feedings can be administered in 2 ways: intermittently, which typically involves feeding over a period of 10 to 20 min every 2 to 3 h, or continuously over 24 h via either a nasogastric or orogastric tube. In healthy-term infants, intermittent bolus feedings trigger a “cyclical pattern of gastrointestinal tract hormones and enteric peptides.” This process not only enhances postprandial splanchnic perfusion but also supports the maturation of gastrointestinal motility [115]. However, functional limitations in the gastrointestinal system of preterm infants, such as delayed gastric emptying, can hinder their ability to handle bolus milk feedings, which may potentially result in feeding intolerance [115]. Moreover, this feeding schedule fluctuates between feeding intervals and fasting periods, potentially challenging the preterm infant’s capacity to sustain metabolic balance and ultimately affecting growth [116].

Continuous feeding is believed to have several benefits for preterm infants such as “enhanced nutrient absorption, optimized energy efficiency, stimulation of enteric motor function, improved growth, and reduced feeding intolerance.” However, continuous feeding may lead to the loss of fat and minerals because these nutrients can adhere to the plastic tubing when slower flow rates are used during the feeding process [117]. In a Cochrane review [116] comparing these 2 methods in preterm infants, no difference in time to achieve full enteral feeds, full oral feedings, growth, feeding intolerance, or incidence of NEC was found. In resource-limited settings, intermittent bolus feedings may be more practical because they only require an intragastric tube and monitoring of individual feeds. In contrast, continuous feeding may necessitate a syringe pump and frequent monitoring, which can be challenging in many hospitals or healthcare centers due to cost and availability constraints [61].

Intermittent bolus feedings can be administered in 2 ways: by syringe (push feed) where milk is “gently pushed into the infant’s stomach using a syringe” or by gravity where milk is “poured into a syringe attached to the tube, and it drips into the infant’s stomach solely due to gravity” [118]. A Cochrane review [118] that considered only 1 trial involving 31 preterm infants concluded that the evidence was insufficient to determine if pump or gravity intermittent bolus feeding had any effect on feeding intolerance, rapid attainment of full enteral feeds, and other adverse outcomes in preterm and LBW infants. In terms of feeding interval of intermittent bolus feeding, a Cochrane review [119] found no effect of 2- and 3-h feeding intervals on time to reach full enteral feedings, feeding intolerance, NEC, or hypoglycemia in preterm infants.

#### Feeding method – oral route

Direct breastfeeding is the ideal oral feeding method for MOM-fed preterm infants; however, this may not be possible in high-risk or acute care settings [120]. Hence, MOM-fed preterm infants may rely on alternative oral feeding methods such as cup feeding, spoon feeding, or bottle feeding. Likewise, DHM-fed and infant formula-fed preterm infants rely on these alternative oral feeding methods. The use of bottle feeding is strongly discouraged in resource-limited settings as ensuring proper care and cleaning of bottles and nipples can be challenging [121]. Cup feeding provides a practical solution as cups are easy to clean and readily available, and thus it is a commonly used alternative feeding method and the recommended alternative oral feeding method in resource-limited settings [121,122]. Cup feeding is also thought to prevent nipple confusion, enhance an infant’s sucking ability in a similar manner to breastfeeding, and facilitate an infant’s ability to self-regulate feeds [120,123]. According to a Cochrane review [124], cup feeding in preterm infants is associated with higher rates of exclusive or predominant breastfeeding compared with bottle feeding at the time of discharge and at 3 and 6 mo after discharge. The review found no differences in the time to reach full oral feeds, duration of feeding, weight gain, length of hospital stays, or episodes of infection per infant with the use of cups or bottles. The reviewed studies also described no evidence of gagging, choking, or aspiration with cup feeding.

#### Nutrition management of preterm infants with oral feeding deficits

Preterm infants, particularly those <32 weeks of gestation, have deficits in sucking and swallowing physiology [11,125,126]. These deficits pose health challenges such as apnea, bradycardia, laryngeal penetration, and airway aspiration during oral feeding attempts warranting a unique set of interventions that may have varying nutritional consequences [9]. In high-income settings, interventions aimed at optimizing feeding safety and comfort include the modifications to infant positioning to feed in a side-lying position, the provision of “paced” feeds by periodically tilting the bottle shaft down to remove the milk from the nipple tip, and the reduction of milk flow rate by using a “slow flow” bottle nipple system [[126], [127], [128], [129], [130], [131]]. These interventions are commonly employed concurrently for most preterm infants due to the relative ease with which they can be applied and the limited negative side effects. Though the evidence testing their effects is limited and their applicability in resource-limited settings is not known, the central theory across all 3 of these interventions is that they promote respiratory-swallow coordination and consequently facilitate cardiopulmonary stability by reducing the stimulus of the bolus that initiates the swallow reflex.

In contrast to these relatively low-risk interventions is the treatment of oropharyngeal swallowing deficits through the provision of thickened liquids, an intervention with significantly greater health implications owing to the pros and cons of the characteristics of the thickener [[132], [133], [134]]. Thickened liquids are primarily postulated to improve preterm feeding by slowing the rate that the bolus flows through the pharynx to enable the infant to more effectively close their airway to prevent penetration and aspiration [135,136]. In the mildest cases, liquids are thickened to a “slightly thick” consistency that targets the thickness of an antireflux formula, with more severe cases requiring thicker liquids such as “mildly thick” or in the most severe cases “moderately thick.” Within the pediatric population, thickened liquids can be formulated by adding varying products to the infant’s milk. Commercial thickeners, such as those with a modified corn starch or gum base, are contraindicated in the preterm infant prior to term gestation due to the risk they pose in causing NEC [137,138]. However, infants may be chronologically mature but the gut and controlling/regulating/adapting apparatus may still be immature, hence the concern of recommending thickeners in preterm-born infants.

Cereal thickeners, such as infant oatmeal, are postulated to pose a lower threat in the development of NEC and yet come with their own myriad of negative effects. One significant implication of these cereals is that they are not effective in thickening human milk, which has enzymes that break it down [132,136]. As such, infants requiring thickeners are frequently switched off human milk and instead provided formula, which can be thickened with cereals, for oral feeds [132]. The negative systemic health implications of this transition are clear, with a poorly understood risk-to-benefit ratio. Other implications of cereal thickeners are related to their detrimental effects on nutrient absorption, which has been shown to be significantly reduced in their presence, and constipation, which often further inhibits oral feeds and inconsistent thickening effects [[139], [140], [141], [142]]. Furthermore, thickened formulas or formulas that are designed to be thickened are not nutritionally complete for preterm infants. Overall, thickened liquids should be considered as a last resort intervention only employed after an interdisciplinary team discussion that has considered the risk-to-benefit ratio amidst the aforementioned negative health implications.

#### Responsive and scheduled feeding

Responsive feeding, also known as cue-based feeding, infant-led, or demand feeding, is defined as feeding in response to “infant visual and auditory cues (or signals)” of hunger and satiety (Table 5). Examples of infant cues indicating readiness to feed include hand–mouth motions, suckling, crying, and mindful awareness [143]. These cues also present as autonomic changes in an increase in heart rate and breathing with increasing alertness and hunger. However, such cues need to be recognized, interpreted, and feeds prepared and implemented by the caregiver in the context of high-risk infant settings, whether it is during the NICU stay or after [9]. Thus, parent/provider characteristics are equally important in ensuring the success of this approach. Additionally, responsive feeding may enhance parents’ and caregivers’ experience and satisfaction in caring for their infants. Conversely, scheduled feeding is defined as feeding prescribed volumes at regularly timed intervals, irrespective of infant cues.

**TABLE 5:** Distinguishing responsive versus scheduled feeding

Responsive feeding	Scheduled feeding
• Infant driven	• Caregiver/provider-driven
• Emphasis is on feeding quality	• Emphasis is on the quantity of feed consumed
• Awareness of an infant’s state of readiness to feed	• Task-oriented without consideration of an infant’s readiness to feed

According to a 2016 Cochrane review [143], when comparing responsive feeding to scheduled feeding of preterm infants during the transition from intragastric feeding to oral feeding, it was found that responsive feeding led to slightly slower rates of weight gain. However, it reduced the time it took for infants to transition from enteral tube to oral feeding. Similarly, a more recent systematic review of preterm and LBW infants found that responsive feeding, when compared to scheduled feeding, resulted in slower hospital weight gain and a reduction in the length of hospital stay [144]. Given the findings on slower weight gain, scheduled feeding rather than responsive feeding may be considered for hospitalized preterm infants <34 weeks of gestation [49]. No recommendations on a particular scheduled feeding interval are available; however, a 2- to 3-h scheduled feeding interval is commonly used with no evidence of harm [49]. Irrespective of the feeding regime provided, the WHO recommends that “emphasis on nurturing and responsive caregiving are crucial and should be implemented to promote the well-being and developmental care of preterm and LBW infants” [49].

### Postdischarge nutrition

At discharge, several nutrient deficits may persist in preterm infants. This is especially evident for those born with extremely low weight, those discharged before reaching their term equivalent age, and those who have encountered multiple morbidities that either increased nutrient requirements or restricted nutrient intake during hospitalization [13,145]. In many instances, there is a requirement for accelerated catch-up growth during the first year of life. However, the precise quantity of additional calories and extra nutrients needed beyond the requirements of full-term infants remains unclear [146].

Exclusive breastfeeding (EBF) is recommended for preterm infants until 6 mo of age [49,147]. There is no clear evidence that an earlier introduction of complementary feeding facilitates more catch-up growth. Continuing EBF until 6 mo is more cost effective than starting complementary feeding earlier. The possibility of contamination during the preparation of complementary foods is also a matter of concern in resource-limited settings. Based on a systematic review [148] of 2 trials involving 207 preterm and LBW infants from India and Honduras, the evidence remains highly uncertain regarding whether there exist any significant differences in terms of mortality, morbidity, growth, and neurodevelopment outcomes between those who were exclusively breastfed for <6 mo and those who were breastfed for 6 mo. In the study conducted by Gupta et al. [149], which involved both EBF and non-EBF preterm infants, introducing complementary feeding at 4 mo of age elevated the likelihood of hospitalization due to infectious diseases.

Human milk fortification after discharge may offer benefits for achieving optimal weight, length, and head circumference growth. Although there are concerns that the extra efforts that fortification after discharge requires impact breastfeeding success, these concerns are unfounded [150]. A Cochrane review [151] of 2 trials, however, did not identify any evidence to support the use of multicomponent fortification of MOM for 3 to 4 mo after hospital discharge. In the review, no significant differences in growth or neurodevelopment at 18 mo were observed between those who received fortified human milk and those who did not. For preterm infants discharged on infant formula, nutrient-enriched formulas such as preterm formula or postdischarge formula, when compared to standard formulas, are associated with improved weight, length, and head circumference gain during infancy. However, there is no evidence of any impact on neurodevelopment or long-term growth outcomes [146,152]. No recent recommendations on human milk fortification postdischarge and the use of nutrient-enriched formulas are available.

### Growth monitoring in the NICU and beyond

Anthropometry is an essential tool for monitoring growth and guiding nutrition support in preterm infants [153,154]. Continued growth monitoring beyond discharge is necessary to tailor feeding to the needs of individual infants so that underfeeding or overfeeding can be avoided. Anthropometry involves the measurement of body weight, length, and head circumference, and occasionally involves the measurement of BMI, skin fold thickness (SFT), and arm and thigh circumference (TC) [154,155]. In most NICUs in high-income settings, the measurement of weight occurs daily whereas the measurements of length and head circumference occur weekly. However, in resource-limited settings, anthropometric measurements may be less frequent due to staff constraints, but the need for longitudinal measurements remains important.

#### Body weight

Body weight measurement is a straightforward and reproducible method commonly used for monitoring the growth and nutritional status of preterm infants [154]. The predominant determinant of weight gain in neonates is the intake of energy, derived from carbohydrates and fats, surpassing the resting energy expenditure with sufficient protein intake [24]. However, body weight measurements are affected acutely by hydration status, and gains may not reflect true growth. Also, the 7% to 20% decrease in birth weight often seen in neonates within the first 3 to 5 d of life is not usually a result of energy deprivation. It is due to the contraction of the extracellular fluid compartment, leading to water losses, although insufficient intake of energy and nutrients, leading to the breakdown of endogenous glycogen, fat reserves, and lean tissue, may play a role [156]. It is anticipated that most infants will return to their birth weight within 7 to 14 d, however, this return to birth weight is dependent on infant, disease, and process factors. Optimizing nutrition and hydration facilitates early recovery from the initial weight loss by reducing both the extent and duration of the weight loss and the time taken to return to birth weight [4,157]. Delayed return to birth weight in conjunction with inadequate nutrient intake may indicate malnutrition because a much higher growth rate that is likely difficult to achieve would be necessary for later catch-up growth [157,158].

Once birth weight is regained, it is recommended to assess weight trends by calculating weight gain velocity with a time interval of at least 5 to 7 d. This approach aids in the early identification of growth faltering and allows for effective monitoring of the response to nutritional interventions [159]. Weight gain velocity calculated as grams per kilogram per day is commonly used because it accounts for the variability in daily measurements. It is suggested that body weight increase rates of 15 to 20 g/kg/d are achievable for infants born between 23 and 36 weeks of gestation and should be the goal [160]. Accurate measurement of weight requires that the infant be weighed nude, and it is preferred that the measurement be taken at the same time each day. An electronic scale that is regularly calibrated should be used, and measures should be recorded to the nearest 0.1 kg. The weight of medical equipment that cannot be removed from the infant during the weighing procedure should be subtracted to improve accuracy [155].

#### Body length

Body length measurement is considered a more sensitive measure of nutritional status than weight because it reflects lean tissue mass and is unaffected by hydration status [154]. Length growth velocity of ∼0.9 to 1.1 cm/wk has been reported in preterm infants [161]. Length is often difficult to accurately measure due to the perceived difficulty in measuring neonates and the use of nonstandard tools such as measuring tape [162]. To accurately measure the length of preterm infants, a rigid length board and 2 examiners are necessary with one holding the infant’s head and the other holding the infant’s legs. To measure length, “the infant should be placed in a supine, fully extended position with knees straightened and feet at right angles to the body, and measurements should be recorded to the nearest 0.1 cm” [155].

#### Head circumference

Head circumference reflects brain size and is thus an essential indicator of neurodevelopment outcomes in preterm infants [163]. Poor head growth in the NICU and after discharge is associated with poor cognitive outcomes [164,165]. Poor head growth may, however, be unrelated to inadequate nutrient intake and may be due to morbidities such as posthemorrhagic hydrocephalus, brain atrophy, or encephalopathy of prematurity [166]. Hence, it is not a sensitive or specific measure of poor growth and nutritional deficits in preterm infants. Head circumference can be further confounded by hydration status and may decrease by 0.5 cm during the first postnatal week due to contraction of the extracellular fluid space [158]. A head circumference growth velocity of 0.9 to 1 cm/wk has been reported in preterm infants [161]. Head circumference should be “measured to the nearest 1 mm with a non-stretch measuring tape at the maximal occipitofrontal circumference.” The largest occipitofrontal circumference of 3 consecutive measurements should be considered [154].

#### Body mass index

BMI (weight in kilograms per length in meters squared) can be a useful anthropometric index for estimating body fatness and for identifying and quantifying disproportionate growth [167]. Preterm infants have been shown to have less fat-free mass, a higher relative amount and distribution of visceral adipose tissue, and a higher percentage of total body fat than their term-born counterparts at term equivalent age [83]. Inadequate accretion of lean mass during NICU hospitalization is associated with suboptimal neurodevelopmental outcomes [168,169]. Compared to other more complex methods such as dual-energy x-ray absorptiometry (DXA), isotope dilution techniques, MRI, total body electrical impedance analysis, or air displacement plethysmography (ADP), BMI can provide a fast and noninvasive way to assess a preterm infant’s quality of weight gain [170]. In a study by Daly-Wolfe et al. [171], BMI was significantly correlated with the percentage of body fat measured by ADP. However, another study by Kiger et al. [172] reported that BMI only accounted for 51% of the variations observed in percent body fat for infants born <32 wk PMA and at <50 wk PMA and 16% of the variability after ≥50 wk PMA. In another study, BMI explained only 27% of the variability in body fat [170]. Thus, uncertainty regarding the usefulness of BMI in preterm infants remains, but serial trends in individual settings may offer some insights into the personalization of feeding strategies.

#### Mid-upper arm and thigh circumference

Mid-upper arm circumference (MUAC) reflects the arm muscle and fat mass composition, with a reduction in measurement indicating a decrease in body muscle and/or fat mass [154]. MUAC measurements are less affected by changes in fluid status and may better reflect actual growth in the presence of edema [154]. MUAC has been observed to increase with gestational age, and a positive correlation with weight, length, and head circumference has been observed in preterm infants [161,173]. MUAC has also been identified as a significant covariate of percentage body fat as it explained 60.4% of the variance in percent body fat in a sample of preterm infants [171]. TC is not a commonly assessed metric in preterm infants. In a study by Ashton et al. [173], significant positive correlations were observed between TC, MUAC, weight, length, and head circumference, and measures were observed to change over time. The utility of MUAC and TC warrants further investigation because it is not clear if these measures are related to body composition or how nutritional intake impacts the rates of change. Preterm infant reference curves for MUAC [161,174] and TC [175] are available. To appropriately measure MUAC and TC in preterm infants, a flexible nonstretchable measuring tape with a width of 1 cm should be used [154].

#### Skinfold thickness

SFT measurements represent a method of assessing the distribution of subcutaneous body fat in the preterm infant [154]. A good correlation between body fat determined by SFT and DXA in term infants at birth and 2 and 4 mo of age has been reported [176,177]. Daly-Wolfe et al. [171] also observed a high correlation between suprailiac skinfold and body fat measurement determined by ADP. In contrast, SFT overestimated total body fat when compared with body water dilution measurements [178] and had poor agreement with DXA and ADP in estimating fat mass [179], and the percentage of body fat determined by SFT was weakly correlated with estimates by isotope dilution [180]. This measure is also affected by the hydration status of infants, does not consider intra-abdominal fat, and assumes that the proportion of total body fat is constant [154]. Reproducibility has also been observed to be low, with an extremely high interobserver error rate. Furthermore, its use can be challenging in extremely preterm infants because of their small size, fragility, and underdeveloped skin, which is prone to bruises [181]. Measurements of SFT involve pinching the skinfolds at specified locations using appropriate calipers and pulling the fold away from the underlying muscle [154]. Specified locations for measurements include the triceps, biceps, subscapular, suprailiac, and quadriceps skinfolds. References for triceps, biceps, subscapular, and suprailiac skinfolds have been described for preterm infants born after 32 weeks of gestation [182,183].

#### Growth curves

The adequacy of growth of preterm infants is assessed and monitored by plotting their weight, length, and head circumference on growth curves, from which specific percentiles and *z*-scores are derived [184]. Percentiles provide information on an infant’s growth measure in comparison to a reference population, whereas the *z*-scores provide information on how much and how far an infant is deviating from the mean or median of the reference population [184]. Growth curves are useful for the assessment of size at birth with infants being categorized as small for gestational age (<10th percentile, −1.28 *z*-score), appropriate for gestational age (10th to 90th percentile, −1.28 to +1.28 *z*-score), or large for gestational age (>90th percentile, >1.28 *z*-score); the assessment of size at hospital discharge with infants at <10th percentile being categorized as having extrauterine growth restriction; and longitudinal monitoring of postnatal growth using serial size measures to determine if growth trajectory is normal or deviating from normal [184]. Longitudinal monitoring of growth patterns over time rather than single 1-time percentile measurements is better for identifying abnormal growth and is recommended [185]. Criteria to define malnutrition have been created by the Academy of Nutrition and Dietetics. This criterion is based on the decline in weight-for-age *z*-scores after the first 2 wk of life. A *z*-score decline of 0.8 to 1.2 is considered mild malnutrition, a decline >1.2 to 2 is considered moderate malnutrition, and a decline >2 is regarded as severe malnutrition [158].

Two types of growth curves are available for assessing the growth of preterm infants: intrauterine and postnatal growth curves. Intrauterine growth curves are created using cross-sectional measurements of birth weight, length, and head circumference from a diverse range of newborns at different gestational ages to reflect in utero growth [186]. Conversely, postnatal growth curves are created using longitudinal growth data from preterm infants as a reflection of their growth patterns after birth [187]. Growth curves may either be created as standards or references. References describe newborn size at various gestational ages and provide insights into how a fetal population grew at a specific location and period. In contrast, standards prescribe how preterm infants should grow, given optimal conditions, aside from their preterm birth [188]. Table 6 [186,187,189,190] provides examples of some growth curves applicable to preterm infants.

**TABLE 6:** Select growth curves for preterm infants

Growth curve	Description
The Fenton growth curve references [186]	• The largest intrauterine growth-based curve references for preterm infants. • Developed in 2013 through a meta-analysis of studies including infants of 22 to 40 weeks of gestation born between 1991 and 2007. • Data were obtained from developed countries including Germany, Italy, United States, Australia, Scotland, and Canada where factors limiting fetal growth may be uncommon. • Data for curve development included 3,986,456 infants for weight, 173,612 infants for head circumference, and 151,527 for length. • Sex-specific curves for birth weight, length, and head circumference were developed. • Curves were developed based on the actual gestational age in weeks and days, as opposed to completed weeks. • Curves begin at 22 weeks of gestation and end at 50 wk PMA. • Validation **was performed for the portion of the curve between 37 and 50 wk by comparing them with the patterns of weight gain in preterm infants** [189]. • The curve was “harmonized with the WHO Growth Standard at 50 weeks to ensure a smoother transition of the preterm infant growth monitoring to the WHO charts.” • A *z*-score and percentile calculator are available for download from http://ucalgary.ca/fenton and on Pedi Tools (http://www.peditools.org/).
The INTERGROWTH-21st preterm postnatal growth curves standards [187]	• Contemporary postnatal growth curve standard created in 2015. • Derived from the longitudinal growth of a cohort of healthy preterm infants across 8 countries, including Brazil, Italy, Oman, United Kingdom, United States, China, India, and Kenya. • Included infants were born to low-risk healthy pregnant women who had accurate pregnancy dating based on first-trimester ultrasonography and showed no signs of intrauterine growth restriction as determined by serial ultrasound assessments. • After birth, the preterm infants in the final cohort received standardized feeding, which primarily involved feedings with fortified human milk. • Infants who experienced complications, like necrotizing enterocolitis, chronic lung disease, sepsis, or any other postnatal conditions that could impact growth, were excluded from the cohort. • The final sample used to create the curves included 201 preterm infants, with only 28 born at a gestational age of <34 wk due to its strict selection. • As such, this curve is only reliable for assessing the postnatal growth of preterm infants with gestational age at birth of 33–36 completed weeks and can be used to assess their growth until 64 wk PMA.
WHO Growth Curves [190]	• The gold standard for assessing the growth of young children from full-term gestational age to 2 y. • Developed based on data from the WHO Multicenter Growth Reference Study (MGRS), a worldwide study conducted during 1997–2003 to create new sex-specific growth curves for assessing growth in infants and young children from birth to 5 y. • Weekly longitudinal growth data from ∼8500 children from Brazil, Ghana, India, Norway, Oman, and United States were used to create the curves. • Included infants were chosen from communities where economic constraints were unlikely to hinder growth. These communities consisted of culturally diverse non-smoking mothers who intended to breastfeed. • All the infants used in the creation of the curves were breastfed for ≥4 mo and continuously breastfed for 12 mo. • Postterm growth monitoring for preterm infants can be accomplished using the WHO Child Growth Standards. Measurements should be plotted based on corrected postnatal age for prematurity until 24 or 36 mo of age. • Exact *z*-score and percentile are available on Pedi Tools (http://www.peditools.org/).

## Conclusions

All reviewed nutrition, feeding, and nutrition strategies (Table 3) are relevant for both resource-abundant and resource-limited settings. Most studies that provided evidence for the recommended strategies were conducted in high-income settings where the levels of mortality and morbidity are generally low. Thus, these strategies are likely to have an even greater impact in resource-limited settings if fully implemented, and the few studies from these settings showed similar findings. However, there is inequitable access to some of the strategies. Although alternative strategies exist, they do not guarantee the same or even close outcomes. For instance, infants unable to feed enterally for prolonged periods in the absence of parenteral nutrition will accrue significant nutritional deficits, yet there are no specific strategies in place to provide additional nutrients to catch up once the infant can feed enterally. Even if such strategies exist, excessive catch-up growth may be a concern for adverse cardiometabolic outcomes, although this remains to be studied in this group of disadvantaged preterm infants. Of all the strategies, the provision of MOM is likely the most cost effective in resource-limited settings, and every effort must be made to support mothers to provide their own milk. Mothers, families, and caregivers should be provided with educational resources on the importance and benefits of exclusively breastfeeding preterm infants while in the hospital and after discharge because of its potential to promote either adequate growth and development and prevent infections and mortality.

## Author contributions

The authors’ responsibilities were as follows – SRJ: conceptualized, designed, and supervised the review; FEB, KEM, CJV, SRJ: analyzed the background literature, drafted, and edited the manuscript; KEM, CJV; provided critical review; SRJ: reviewed, revised, and verified the manuscript; and all authors: read and approved the final manuscript.

## Funding

The authors reported no funding received for this study.

## Conflict of interest

The authors have no competing interests to declare that are relevant to the content of this article. Dr Valentine serves as Chief Medical Officer for Kate Farms Nutrition, but they had no input, nor do they manufacture Infant nutrition products. This work was a result of her academic role.
